# PRevalence of the Eosinophilic Phenotype Among SeveRE asthma patients in Lebanon: results of the PREPARE study

**DOI:** 10.1186/s13223-023-00815-1

**Published:** 2023-09-08

**Authors:** Wajdi Abi Saleh, Zuhair Alameh, Zeina Aoun Bacha, Joudy Bahous, Pierre Bou Khalil, Zahia Chahine, Hassan Chami, Georges Dabar, Hassan Dheiny, Alfred Dib, Dany farhat, Carla Irani, Georges Juvelekian, Nadim Kanj, Bassam Mansour, Moussa Riachi, Mirna Waked, Mohamad Yassine, Carole Youakim, Salah Zeinedine, Fares Zaitoun

**Affiliations:** 1 Department of Pulmonary and Critical Care Medicine, Respiratory Institute, Cleveland Clinic Foundation, Cleveland, OH USA; 2 Pulmonology Practice, Private Practice, Beirut, Lebanon; 3 Department of Pulmonology and Critical Care, Hôtel-Dieu De France Medical Center (UMC), Saint-Joseph University (USJ), P.O. Box 2064-6613, Beirut, 1104 2020 Lebanon; 4 Department of Internal Medicine, Division of Pulmonary and Critical Care Medicine, Saint George Hospital University Medical Center, Beirut, Lebanon; 5 Department of Internal Medicine, Division of Pulmonary and Critical Care, American University of Beirut Medical Center, Beirut, Lebanon; 6 Division of Pulmonary and Critical Care, Johns Hopkins University School of Medicine, Baltimore, MD USA; 7 Department of Pneumology, NINI Hospital, Tripoli, Lebanon; 8 Respiratory Diseases, Allergies and Sleep Medicine, Private Practice, Beirut, Lebanon; 9 Department of Internal Medicine, Division of Pulmonology, Sacre-Coeur Hospital, Baabda, Lebanon; 10 Hammoud Hospital, Sidon, Lebanon; 11 Department of Internal Medicine & Clinical Immunology at Hôtel-Dieu de France, St Joseph University, Beirut, Lebanon; 12 Department of Pulmonary and Critical Care Medicine, Zahraa Hospital, Beirut, Lebanon; 13 Department of Internal Medicine, Division of Pulmonary, Mount Lebanon Hospital Balamand University Medical Center, Beirut, Lebanon; 14 Department of Pediatrics and Adolescent Medicine, American University of Beirut Medical Center, Beirut, Lebanon

**Keywords:** Severe asthma, Eosinophilic phenotype, Prevalence, Lebanon

## Abstract

**Background:**

The prevalence of eosinophilic asthma in Lebanon, one of the most severe phenotypes among severe asthma, is not known. This study aimed at determining the prevalence of the eosinophilic phenotype defined as an eosinophil count ≥ 300 cells/mm^3^ among severe asthma patients in Lebanon.

**Methods:**

The Lebanese Chapter of the PREPARE study was a national, multicenter, cross-sectional observational study. Patients aged ≥ 12 years with severe asthma were identified and prospectively enrolled during clinic visits and completed the Global Initiative for Asthma (GINA) assessment of asthma control questionnaire. Patients’ health characteristics were collected from medical records and blood samples were obtained for measurement of serum IgE levels and blood eosinophils count.

**Results:**

Overall, 101 patients (with mean age of 46.3 ± 17.0 years and 73.27% females) with severe asthma were included and, among them, 37% had eosinophilic phenotype, 67.3% had atopic phenotype with IgE > 100 IU/mL and 25.7% patients had overlapping atopic and eosinophilic phenotypes. Close to 80% had late-onset asthma, beyond 12 years of age, and around 85% had at least one severe exacerbation in the 12 months prior to study enrolment. The majority of participants [64.4%] had uncontrolled asthma, 24.7% had partially controlled symptoms and 10.9% had controlled symptoms. 19.8% of participants were on chronic oral corticosteroids, 78.2% had short course treatment of corticosteroids and all were prescribed a combination of inhaled corticosteroids and long-acting beta-agonist.

**Conclusions:**

The majority of patients with severe asthma were uncontrolled of which 37% present with an eosinophilic phenotype, which should be taken into consideration for better management of these patients in view of the novel phenotype-specific therapeutic options.

## Background

Asthma is a leading cause of morbidity with a global prevalence of approximately 300 million and is expected to increase to 400 to 450 million people worldwide by 2025 [1]. In Lebanon, the prevalence of asthma has been estimated at 9% in 2014 [2] and at 6.7% in 2021 [3].

Despite the availability of multiple therapeutic options, 5–10% of asthmatic patients experience severe disease associated with substantial morbidity.

Severe asthma poses a great burden on the healthcare system [4, 5] that account for up to 50% of the total asthma-associated healthcare costs [6, 7]. Patients with severe asthma suffer from greater levels of anxiety and depression and report markedly worse quality of life (QoL) indicators than patients with mild or moderate asthma [8].

Severe asthma is currently considered a heterogeneous disease, with various phenotypes, owing to the variety of inflammatory, clinical and functional characteristics that it can present with. One of the most studied phenotypes is severe eosinophilic asthma [9, 10]. Eosinophils have long been recognized as an important element in asthmatic inflammation. This persistent airway inflammation is partly responsible for the high frequency of exacerbations seen in severe asthma [11]. Patients with severe asthma accompanied with a high eosinophil concentration require greater healthcare resource use, overall greater disease management costs and have a much lower QoL than those who do not present with eosinophilia [12–14].

Peripheral blood eosinophil counts, as high as 400 cells/mm^3^, have been linked to increased asthma exacerbations, and adult-onset asthma patients with blood eosinophil counts ≥ 300 cells/mm^3^ is a distinct phenotype of severe asthma characterized by frequent exacerbations and poor prognosis [15, 16]. Additionally, studies indicate that asthma patients with blood eosinophil levels ≥ 300 cells/mm^3^ benefit from targeted treatment of eosinophilic inflammation [17, 18].

Although eosinophilic inflammation of the airways has been classically associated with allergic asthma [19] there is evidence that eosinophilia is present in the airways of severe asthmatic patients without allergic disease [20]. Contrary to early-onset eosinophilic asthma, adult-onset eosinophilic asthma frequently develops in the absence of allergen-dependent activation of Th2 lymphocytes, which suggests a mechanism of eosinophilic inflammation other than allergy [21]. Late-onset asthmatics have also been found to present a lower rate of skin prick sensitization, indicating that eosinophilic airway inflammation and atopy are not fully concordant [22]. Furthermore, asthma patients may present with atopy (i.e., elevated immunoglobulin E [IgE] levels), without sensitization to common inhaled allergens or allergic etiology [23].

Severe asthma includes multiple distinct and at time overlapping phenotypes that need to be well characterized in order to guide treatment. Yet, the knowledge of the prevalence of eosinophilia among severe asthma patients in many countries is still limited and the prevalence of the atopic phenotype within the severe asthma population is scarce.

The overall objectives of the Lebanese Chapter of the PRevalence of the Eosinophilic Phenotype Among SeveRE Asthma Patients.

(PREPARE) study were to estimate the prevalence of the eosinophilic and atopic phenotypes among severe asthma patients in Lebanon and assess asthma control, among this population.

## Methods

### Study design and participants

#### Study design

This was a multicenter, observational, descriptive study with a cross-sectional design. The Lebanon chapter of the multinational PREPARE study relied on data collection forms used globally, for uniform and consistent collection of data. Data were collected from 21 centers, to assess the prevalence of eosinophilic and atopic phenotypes among severe asthma patients in Lebanon.

The study was approved by the Institutional Review Board (IRB) of the following institutions: American University of Beirut Medical Center (D2287R00140/BIO-2020-0301), Clemenceau Medical Center (ERRC/CS/03/2019), Saint George Hospital University Medical Center (IRB-REC/O/033-22/2320), Hôtel-Dieu De France Hospital (CEHDF 1700), Hammoud Hospital, Sacre-Coeur Hospital and Al Zahraa Hospital.

All participants signed an informed consent form (E2014131); minor patients gave consent to participate, validated by a parental consent form.

#### Participants

Patients aged 12 years and older with severe asthma, as defined by the GINA 2018 Guidelines (GINA, 2018) were identified and invited to participate during routine clinical visits, until around 100 patients were enrolled. Eligible patients provided written consent, and agreed to provide a blood sample for IgE and eosinophil levels determination as part of routine care, and were diagnosed with severe asthma for at least 1 year as defined by treatment with guidelines-suggested medications for GINA steps 4–5 asthma. Patients with chronic obstructive pulmonary disease or other acute or chronic respiratory conditions that, in the investigator’s opinion, would limit the patient’s ability to participate in this study, and patients receiving biologics treatment for severe asthma were excluded.

### Data collection

Participants completed the GINA assessment of asthma control questionnaire and provided a blood sample to quantify total serum IgE levels and complete differential blood count, including eosinophils. Data on atopy history, skin prick tests, specific IgE and blood works in the last 5 years blood, in addition to comorbidities (rhinitis, nasal polyps, atopic dermatitis), current pharmacological treatment and the rate of severe asthma exacerbations in the 12 months prior to study entry were all extracted from the patients’ medical records. Current pharmacological treatment for asthma includes chronic oral corticosteroids (OCS), inhaled corticosteroids (ICS)/ long-acting beta-agonist (LABA) fixed dose combination, and other current maintenance therapies.

### Study objectives

The primary objective of this study was to determine the prevalence of the eosinophilic phenotype, defined as an eosinophil count ≥ 300 cells/mm^3^ among patients with severe asthma in Lebanon. Secondary objectives included characterization of the demographic features, disease control, exacerbations frequency and pharmacological treatment of patients with severe asthma with eosinophilic and atopic phenotypes.

### Study definitions

Asthma was classified as early-onset (before 12 years of age) and late-onset (after 12 years of age). Severe asthma exacerbation was defined according to the American Thoracic Society/European Respiratory Society (ATS/ERS) statement on Asthma Control [10]. The eosinophilic phenotype was defined as blood eosinophil ≥ 300 cells/mm^3^ and the atopic phenotype as serum IgE > 100 IU/mL [24]. Patients with overlapping phenotypes were also reported. Another cut-off of blood eosinophil count ≥ 150 cells/mm^3^ among patients with severe asthma was also assessed.

Asthma control was assessed using the GINA definition and categorized as good control, partial control or uncontrolled [25].

Oral CS burst refers to the use of an intravenous or oral CS for at least three days or to the use of a single intramuscular CS dose. For patients on maintenance oral corticosteroids, at least double the existing maintenance dose for at least three days should be considered.

### Statistical analysis

A sample size of 100 patients was considered to provide sufficient precision to estimate the primary outcome: the prevalence of patients with blood eosinophil count ≥ 300 cells/mm^3^. The precision was assessed based on the expected half-width of the 95% confidence interval around the observed proportion within the country, i.e., the distance between the observed proportion and the upper and lower 95% confidence limits. Descriptive statistics were performed according to the type of criterion. Baseline characteristics and outcomes were described as mean and standard deviation, for continuous variables; and as percentages, two-sided 95% CI for categorical variables.

The primary dependent variable was asthma phenotype categorized as eosinophilic, atopic and overlapping atopic/eosinophilic.

The association of the severe asthma phenotypes with the various characteristics was examined using One-way analysis of variance (ANOVA) for continuous variables (age, body mass index (BMI) and Chi square test or the Fisher’s exact test for categorical variables (smoking status and treatments). A sensitivity analysis was performed to correlate the prevalence of an eosinophilic phenotype, an atopic phenotype, or the overlap of both, as well as asthma control levels and exacerbations with age and BMI categories, smoking status and pharmacological treatment. All analyses were performed using Statistical Analysis Software (SAS) 9.4 or higher. A two-tailed P value < 0.05 was considered statistically significant.

## Results

### Disposition of patients

Participants were enrolled between March and October 2021. A total of 103 patients were identified at 21 centers, over a period of 7.7 months. Of those, two patients did not meet eligibility criteria: one patient diagnosed with severe asthma in less than a year of study initiation while the other had no available information in the database related to suffering from severe asthma. Therefore, only 101 patients were included in this study.

### Demographic and other characteristics

The large majority of patients in this study were female (74 [73.3%]), aged between 12 and 77 years old, with an average age of 46.3 ± 17.0. Table 1 displays the distribution of patients in the pre-defined age categories, educational level, insurance coverage and smoking status, with details on number of cigarettes per day, numbers of years of smoking and time since quitting for former smokers. Smokers were categorized as never-smokers; former smokers, and current smokers. The majority of patients achieved a graduate education level [34.7%], had health insurance [46.5%] and never smoked [60.4%]. BMI categories were defined according to the World Health Organization (WHO) [26].

**Table 1:** Socio-demographics and smoking status

	Study population N = 101 N (%)
Age, in years	
[12–34]	25 (24.8%)
[34–47]	24 (23.8%)
[47–60]	25 (24.8%)
≥ 60	27 (26.7%)
Educational level	
Elementary	22 (21.8%)
High school	19 (18.8%)
Graduate	35 (34.7%)
Post-graduate	24 (23.8%)
Insurance coverage	
Full	47 (46.5%)
Partial	30 (29.7%)
None	23 (22.8%)
Smoking status	
Never	61 (60.4%)
Former	22 (21.8%)
Number of cigarettes per day	15.6 ± 9.2
Number of years of smoking	16.2 ± 11.4
Number of years since quitting	10.9 ± 9.2
Current	8 (17.8%)
Number of cigarettes per day	13.1 ± 11.4
Numbers of years of smoking	17.3 ± 12.1
BMI categories	N (%)
Severely underweight (< 16.5 kg/m^2^)	1 (1.0%)
Underweight (< 18.5 kg/m^2^)	2 (2.0%)
Normal weight ([18.5–25 kg/m^2^])	35 (34.7%)
Overweight ([25–30 kg/m^2^])	35 (34.7%)
Obese (≥ 30 kg/m^2^)	28 (27.6%)

BMI: body mass index; SD: standard deviation

### Prevalence of the eosinophilic and atopic phenotypes

**Fig. 1:**
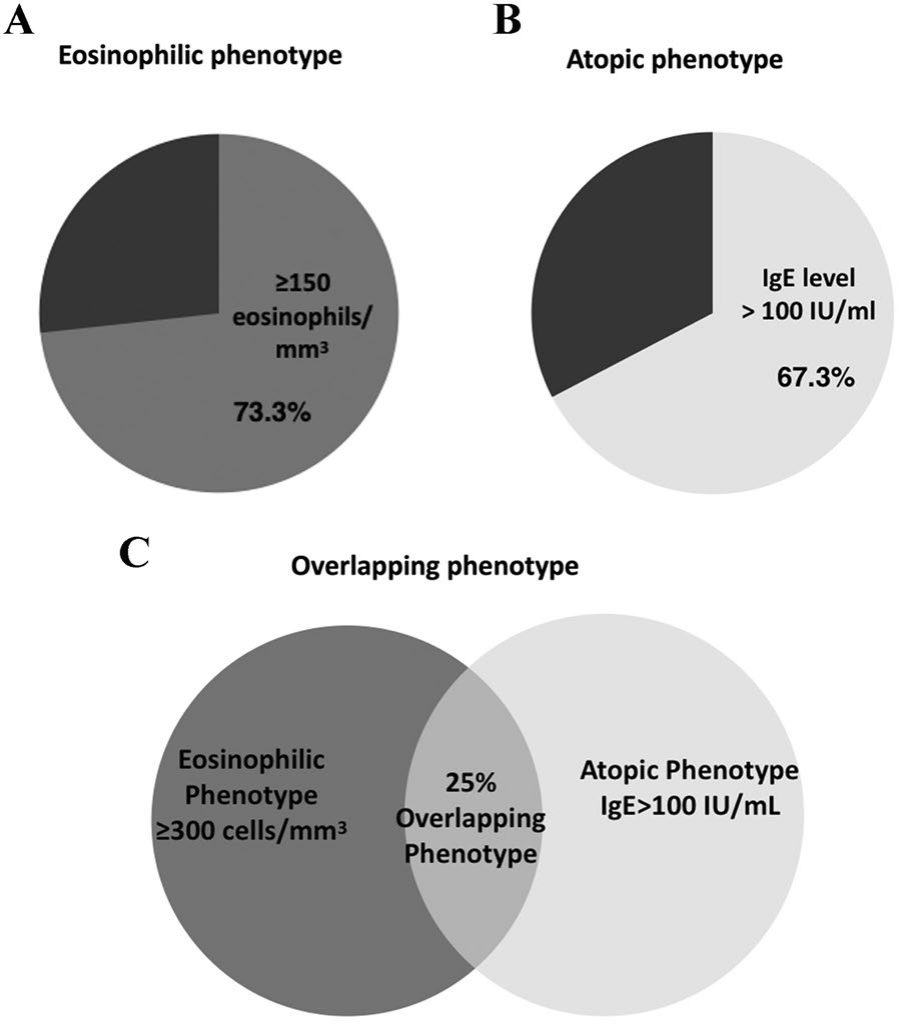
Prevalence of patients with eosinophilic and atopic phenotypes. Prevalence of patients with **A** eosinophilic phenotypes with an eosinophil count ≥ 300 cells/mm^3^ or ≥ 150 cells/mm^3^; **B** atopic phenotype with an IgE level > 100 IU/ml and; **C** proportion of patients with overlapping phenotypes

Among 101 included patients, a total of (74 [73.3%]) patients had a blood eosinophil count ≥ 150 cells/mm^3^, and only (37 [36.6%]) patients had an eosinophilic phenotype with an eosinophil count ≥ 300 cells/mm^3^ (Fig. 1A). 68 [67.3%] presented with an atopic phenotype (IgE level > 100 IU/mL) (Fig. 1B), while (26 [25.7%]) had overlapping atopy and eosinophilia (eosinophil count ≥ 300 cells/mm^3^ and IgE level > 100 IU/mL) (Fig. 1C).

Total serum IgE levels were 488 ± 921.8 IU/mL, absolute eosinophil counts were 393.3 ± 235.4 cells/mm^3^ (Table 2).

**Table 2:** Eosinophil count and IgE level

	Study population N = 101
Prevalence of eosinophilic phenotype	
Blood Eosinophil count ≥ 300 cells/mm^3^	37 (36.6%)
Absolute blood eosinophil count	
Mean ± SD	566.1 ± 221.7
Range	300.0–1242.0
Median	520.00
Blood eosinophil count ≥ 150 cells/mm^3^	
Absolute blood eosinophil count	74 (73.3%)
Mean ± SD	393.3 ± 235.4
Range	150.0 – 1242.0
Median	299.8
Total serum IgE (IU/mL)	
Mean ± SD	488.0 ± 921.8
Range	5.0 – 6220.0
Median	173.00
Prevalence of atopic phenotype*	68 (67.3%)
Serum IgE levels, in IU/mL	
Mean ± SD	689.9 ± 1053.8
Range	102.1 – 6220.0
Median	302.5
Prevalence of overlapping atopy and eosinophilia	26 (25.7%)
Serum IgE levels, in IU/mL	811.5 ± 1078.1
Blood eosinophil count, in cells/mm^3^	610.3 ± 235.2

IgE: immunoglobulin E; SD: standard deviation

*Total serum IgE > 100 IU/mL

### Demographic and clinical characteristics of severe asthma patients

Patients have had asthma symptoms for 208.7 ± 158.9 months and have been diagnosed with asthma for 183.0 ± 153.0 months. Among 101 patients, close to 80% patients had late-onset asthma after the age of 12 years old while a total of 85 patients [84.2%] had at least one severe exacerbation in the 12 months leading up to study entry. Spirometry data were collected for patients who had a spirometry test in the year leading up to study entry. Out of 101 patients, only 22 (21.78%) had available spirometry results. On average, the most recent tests date back to 3.58 ± 4.15 months with a median of 2.30 months. Spirometry results are summarized in Table 3.

**Table 3:** Spirometry results

	N = 22		
	Mean ± SD	Range	Median
Forced vital capacity			
Pre-bronchodilator FVC	3.11 ± 0.88	1.4–4.6	3.3
% of the predicted value	74.24 ± 31.00	1.0–110.0	85.0
Post-bronchodilator FVC	3.39 ± 0.78	1.6–4.6	3.6
% of the predicted value	75.13 ± 33.00	1.0–107.0	87.0
Forced expiratory volume in 1 s			
Pre-bronchodilator FEV1	2.41 ± 0.78	0.9–3.6	2.5
% of the predicted value	80.88 ± 20.07	49.0–127.0	81.0
Post-bronchodilator FEV1	2.66 ± 0.69	1.1–3.5	2.9
% of the predicted value	85.67 ± 18.51	56.0–122.0	89.0
Ratio of FEV1/FVC			
Pre-bronchodilator FEV1/FVC	72.23 ± 21.79	9.6–111.0	73.5
Post-bronchodilator FEV1/FVC	74.21 ± 21.55	9.7–105.0	80.2
Forced expiratory flow at 25–75%	49.93 ± 32.26	0.0––109.0	53.0

FEV1: forced expiratory volume in 1 s; FVC: forced vital capacity; SD: standard deviation

### GINA assessment of asthma control

The majority of patients (65 [64.4%]) had uncontrolled asthma, 11 patients [10.9%] had controlled symptoms and 25 patients [24.7%] had partially controlled symptoms.

### History of atopy and asthma comorbidities

A total of (67 [66.3%]) patients had a history of atopy. Around (41 [83.7%]) out of the 49 patients showed positive results for skin prick test, (29 [90.6%]) out of the 32 patients for aeroallergens, and 21 cases [72.4%] out of 30 patients had a positive specific IgE test.

The majority of patients presented comorbidities [78.2%]: rhinitis (48 [60.8%]), atopic dermatitis 12 [15.2%], nasal polyps (11 [13.9%]), asthma associated with non-steroidal anti-inflammatory drugs (NSAIDs; 5 [6.3%]) and other comorbidities (37 [46.8%]). The other associated morbidities included metabolism and nutrition disorders in 17 (16.8%) patients, gastrointestinal disorders in (14 [13.9%]) patients, vascular disorders in (13 [12.9%]) patients and other less frequent morbidities.

### Prescribed medications for asthma

Asthma treatment in the 12 months prior to study enrollment included chronic OCS, burst/short course corticosteroids, ICS + LABA. Out of the 101 study patients, the majority of patients (79 [78.2%]) needed burst/short course treatment of corticosteroids to up to 10 times over 12 months with an average of 2.3 ± 1.5 per year. All patients were prescribed ICS + LABA (Table 4). Additional maintenance treatments were also prescribed to 82 patients (82.8%) (leukotriene receptor antagonists, inhaled anticholinergics, glucocorticoids, systemic antihistamines) mostly in the form of tablets (88 [50.6%]), (52 [29.9%]) inhalers and nebulizers (28 [16.09%]) and other formulations.

**Table 4:** Prescribed medications for asthma

	Study population N = 101
OCS	N (%) 20 (19.8%)
Corticosteroids burst treatment/short course	N (%) 79 (78.2%)
ICS/LABA	N (%) 101 (100.0%)
Active substances	N (%)
Budesonide/Formoterol	57 (56.4%)
Fluticasone/Salmeterol	17 (16.8%)
Fluticasone/Vilanterol	8 (7.9%)
Beclomethasone/Formoterol	7 (6.9%)
Other	12 (11.9%)
Total daily ICS dose	N = 100
Low dose	0 (0.0%)
Medium dose	34 (34.0%)
High dose	66 (66.0%)
Time since initiation of ICS/LABA, in years	
Mean ± SD	4.5 ± 4.3
Range	0–19.2
Median	2.9
	N = 82
Other maintenance treatments	N (%)
Leukotriene receptor antagonists	58 (70.7%)
Anticholinergics	36 (43.9%)
Glucocorticoids	27 (32.9%)
Systemic anti-histamines	20 (24.4%)

ICS: inhaled corticosteroids; LABA: long-acting beta-agonist; OCS: chronic oral corticosteroids; SD: standard deviation

### Association of asthma phenotype and patient characteristics

Table 5 displays the association between the different asthma phenotypes previously described and patient characteristics. This analysis showed that eosinophilic phenotype was the highest among former smokers (5.3% ± 8.0), compared to never smokers and active smokers (P < 0.05), while the prevalence of atopic phenotype was the highest in never smokers compared to the remaining categories (P < 0.01). Asthma was poorly controlled in obese patients. Recurrent exacerbations were most present in overweight patients and those under a combination of OCS + ICS + other pharmacological treatments (P < 0.01).

**Table 5:** Association between asthma phenotypes and age, BMI, smoking status and pharmacological treatment

Variable			Eosinophilic phenotype ≥ 300 cells/mm^3^	Atopic phenotype	Atopy + eosinophilia	Eosinophilic phenotype ≥ 150 cells/mm^3^	Asthma control			Asthma exacerbations
						n (%)	Good	Partial	Uncontrolled	
Age in years	[12–34]	N = 25	11 (44.0%)	20 (80.0%)	11 (44.0%)	18 (72.0%)	4 (16.0%)	5 (20.0%)	16 (64.0%)	21 (84.0%)
	[34–47]	N = 24	9 (37.5%)	11 (45.8%)	4 (16.7%)	17 (70.8%)	3 (12.5%)	5 (20.8%)	16 (66.7%)	19 (79.2%)
	[47–60]	N = 25	7 (28.0%)	18 (72.0%)	4 (16.0%)	17 (68.0%)	3 (12.0%)	5 (20.0%)	17 (68.0%)	20 (80.0%)
	≥ 60	N = 27	10 (37.0%)	19 (70.4%)	7 (25.9%)	22 (81.5%)	1 (3.7%)	10 (37.0%)	16 (59.3%)	25 (92.6%)
			*P* = 0.706	*P* = 0.065	*P* = 0.084	*P* = 0.711		*P* = 0.636		*P* = 0.520
BMI in kg/m^2^	Underweight	N = 3	2 (66.7%)	2 (66.7%)	2 (66.7%)	2 (66.7%)	1 (33.3%)	0 (0.0%)	2 (66.7%)	2 (66.7%)
	< 18.5									
	Normal range	N = 35	17 (48.6%)	25 (71.4%)	12 (34.3%)	27 (77.1%)	3 (8.6%)	10 (28.6%)	22 (62.9%)	28 (80.0%)
	[18.5–25]									
	Overweight	N = 35	10 (28.6%)	21 (60.0%)	6 (17.1%)	25 (71.4%)	4 (11.4%)	9 (25.7%)	22 (62.9%)	31 (88.6%)
	[25–30]									
	Obese	N = 28	8 (28.6%)	20 (71.4%)	6 (21.4%)	20 (71.4%)	3 (10.7%)	6 (21.4%)	19 (67.9%)	24 (85.7%)
	≥ 30									
			*P* = 0.164	*P* = 0.744	*P* = 0.129	*P* = 0.947		*P* = 0.856		*P* = 0.522
Smoking status	Never	N = 61	28 (45.9%)	42 (68.9%)	21(34.4%)	47 (77.1%)	6 (9.8%)	12 (19.7%)	43 (70.5%)	54 (88.5%)
	Former	N = 18	6 (27.3%)	15 (68.2%)	4 (18.2%)	18 (81.8%)	2 (9.1%)	7 (31.8%)	13 (59.1%)	18 (81.8%)
	Current	N = 22	3 (16.7%)	11 (61.1%)	1 (5.6%)	9 (50.0%)	3 (16.7%)	6 (33.3%)	9 (50.0%)	13 (72.2%)
			*P* = 0.046*	*P* = 0.824	*P* = 0.029*	*P* = 0.055		*P* = 0.457		*P* = 0.219
Pharmacological treatment	ICS only	N = 6	3 (50.0%)	4 (66.7%)	2 (33.3%)	4 (66.7%)	2 (33.3%)	1 (16.7%)	3 (50.0%)	2 (33.3%)
	OCS + ICS	N = 13	4 (30.8%)	8 (61.5%)	3 (23.1%)	11 (84.6%)	2 (15.4%)	3 (23.1%)	8 (61.5%)	12 (92.3%)
	ICS + Other	N = 14	4 (28.6%)	8 (57.1%)	3 (21.4%)	8 (57.1%)	5 (35.7%)	4 (28.6%)	5 (35.7%)	3 (21.4%)
	OCS + ICS + Other	N = 68	26 (38.2%)	48 (70.6%)	18 (26.5%)	51 (75.0%)	2 (2.9%)	17 (25.0%)	49 (72.1%)	68 (100.0%)
			*P* = 0.810	*P* = 0.714	*P* = 0.955	*P* = 0.389		*P* = 0.004**		*P* < 0.001***

Chi-square or Fisher’s exact was performed depending on the expected counts

*P < 0.05, **P < 0.01 and ***P < 0.001

BMI: body mass index; ICS: inhaled corticosteroids; OCS: oral corticosteroids

## Discussion

The PREPARE study was an observational, cross-sectional and multicenter study that aimed primarily at reporting the prevalence of the eosinophilic phenotype among Lebanese patients with severe asthma, aged 12 years and beyond enrolled in 21 medical centers.

The eosinophilic phenotype, defined as an eosinophil count ≥ 300 cells/mm^3^, was identified among 37% of Lebanese patients with severe asthma. This matches regional and global prevalence figures for the prevalence of this phenotype in severe asthma patients. In Saudi Arabia, 45% of patients with severe asthma had the eosinophilic phenotype and half of them had an overlapping atopic phenotype [24]. In Japan, 34% of patients with severe asthma had eosinophilic asthma [27]. Prevalence of Brazilian patients with eosinophilic phenotype was reported at 40.0%, with a high overlap with atopic asthma [28].

Close to 80% of the patients had late-onset asthma; i.e., beyond the age of 12 years old, and, on average, patients in this study were diagnosed with severe asthma for 15 ± 12 years.

Late-onset asthma has been associated with various risk factors of which gender, BMI and smoking status, which drive airway inflammation in asthma [29]. In this study, most patients were female, which is in accordance with the literature that showed that ovarian hormones, contrary to testosterone, enhance innate and adaptive immune response leading to airway inflammation [24, 28, 30]. In addition, around half the patients presented with high BMI of which 35% of patients were overweight and 28% suffered from obesity. The majority of patients were never smokers [60%] and only 18 patients were current active smokers. Only 11% of patients had controlled asthma symptoms during the month leading up to study entry while the majority of patients [65%] had uncontrolled symptoms. These figures are also similar to those reported in Saudi Arabia [24]. Importantly, over 80% of study patients experienced at least one severe asthma exacerbation in the 12 months prior to study entry, and close to 79% needed burst treatment of corticosteroids. Since weight loss [31] and smoking cessation [32] has been shown to improve asthma-related quality of life, asthma control, and lung function, these findings shed the light on the importance of lifestyle changes in order to attenuate the induced asthma exacerbation and improve asthma management outcome.

Furthermore, the highest rate of exacerbations occurred among overweight patients, further underscoring the importance of weight control among asthma patients [24, 31]. While the high level of proinflammatory adipokines in obesity was reported to be associated with airway inflammation leading to asthma in obesity [33], Sideleva et al., showed a direct effect of adipokines causing airway reactivity rather than airway inflammation [34]. The mechanism relating the two diseases remains to be elucidated.

Besides, uncontrolled severe asthma poses a major economic burden on the healthcare system [4, 5] and account for up to half the total asthma-associated healthcare costs [6, 7] a situation that cannot be handled especially in the current critical economic situation in Lebanon.

In accordance with the GINA 2021 updated recommendations for severe asthma treatment, 60% of patients were on formoterol combined to ICS while 71% were also on montelukast, a leukotriene receptor antagonist endorsed in GINA Step 5 asthma management [35].

Despite the adequate treatment application and intensification, the majority of patients had uncontrolled asthma. Hence, the importance of severe asthma classification and the application of targeted phenotype-specific treatments. For atopic asthma, many monoclonal antibodies were developed and showed potential effects: Omalizumab [36] and Dupilumab [37] are both monoclonal antibodies that bind to IgE and IL-4Rα respectively, and may be treatment options when atopic asthma is persistent and inadequately controlled by ICS.

Regarding eosinophilic asthma, several biologics targeting IL-5, mepolizumab [38] or its receptor, benralizumab [39] have recently been approved and showed reduced asthma exacerbation. Hence, the importance of identifying the prevalence of each asthma phenotype in Lebanon for a better rate of asthma control.

This study presented some limitations. First, the cross-sectional nature does not permit to establish causality between clinical asthma characteristics and variables reported in the study (age, BMI, treatment pattern, etc.). Second, the relatively small sample size (further reduced by the exclusion of patients on biologics) might not be representative of the whole population with severe asthma. Third, patients who were on biologic treatment were not included in this study. Patients were enrolled in 21 different sites covering all the regions in Lebanon with a representative sample size adequate for each site though. While the updated GINA 2022 guidelines recommend biologic therapy for eligible asthma patients [25], this observational study did not look into patient eligibility for approved biologics or into the availability of these expensive drugs in Lebanon, similarly to what was reported in Brazil [40].

## Conclusions

In conclusion, this study suggests that a substantial proportion of severe asthma patients in Lebanon present the eosinophilic phenotype and have poorly controlled disease even when treated with medium and high doses of ICS/LABA; they frequently required OCS maintenance and experienced a high frequency of exacerbation. This study highlights the importance of identifying and evaluating this group of patients for phenotype-specific treatment strategy.

## Data Availability

The datasets used and/or analysed during the current study are available from the corresponding author on reasonable request.

## Abbreviations

ANOVA: One-way analysis of variance
ATS/ERS: American Thoracic Society/European Respiratory Society
BMI: Body Mass Index
GINA: Global Initiative for Asthma
IRB: Institutional Review Board
ICS: Inhaled corticosteroids
IgE: Immunoglobulin E
LABA: Long-acting beta-agonist
NSAIDs: Non-steroidal anti-inflammatory drugs
OCS: Chronic oral corticosteroids
SD: Standard deviation
QoL: Quality of life
UMC: Hôtel-Dieu De France Medical Center
